# Linear Neck Pain and Prolonged Cough Caused by Takayasu Arteritis

**DOI:** 10.7759/cureus.27227

**Published:** 2022-07-25

**Authors:** Yoji Hoshina, Jumpei Kojima, Yu Li, Yusuke Hirota, Takanori Uehara, Masatomi Ikusaka

**Affiliations:** 1 General Medicine, Chiba University Hospital, Chiba, JPN; 2 Neurology, University of Utah, Salt Lake City, USA; 3 Diabetology, Endocrinology and Metabolism, Tokyo Medical University Hachioji Medical Center, Tokyo, JPN

**Keywords:** ulcerative colitis, prolonged cough, neck pain, vasculitis, takayasu arteritis

## Abstract

The clinical manifestations of Takayasu arteritis (TA) greatly vary, and this ultimately leads to a delay in diagnosis. We describe a case of TA presenting with two coexisting rare symptoms of linear neck pain and prolonged cough. A 28-year-old Japanese female with a six-month history of ulcerative colitis presented with recurrent left neck pain, cough, and fever. The neck pain and fever started five months ago. Her symptoms briefly improved with nonsteroidal anti-inflammatory drug therapy, but eventually recurred one month prior to her latest presentation to the hospital, which was accompanied by a dry cough. Physical examination revealed a blood pressure discrepancy, with systolic blood pressure being >10 mmHg lower in her left arm than in her right arm, a bilateral carotid bruit, a weak left radial pulse and radio-radial delay without coolness in the upper extremities, and linear pulsatile tenderness in her left neck along the common carotid artery. No supraclavicular or infraclavicular bruit was noted. The erythrocyte sedimentation rate was elevated at 66 mm/hour. After obtaining the images from a contrast-enhanced computed tomography, she was diagnosed with TA. All her symptoms improved with prednisone therapy. Notably, neck pain and cough are both late-stage symptoms of TA, which are seen in 9.7% and 1.5% of patients, respectively. Although her unspecific symptoms could have been easily misdiagnosed, the recurring exacerbation of symptoms warranted careful attention to a focused physical examination.

In conclusion, neck pain and cough are both uncommon presentations of TA, which may lead to physicians underdiagnosing it. It is important to recognize neck pain and cough as presenting complaints in patients with TA.

## Introduction

Takayasu arteritis (TA) is a large vessel vasculitis that primarily affects the aorta and its main branches [1]. Women are predominantly affected with an age of onset usually between 10 and 40 years [2]. The condition has a worldwide distribution, with the greatest prevalence in Asia, especially in Japan [2]. It is characterized by a chronic, waxing, and waning clinical course [2]. Early symptoms are typically non-specific and late symptoms are caused by the narrowing of the affected arteries [3]. Most patients exhibit an insidious presentation, and the diagnosis of TA can be delayed for months or even years, especially in patients presenting with uncommon symptoms [3]. We describe a case of TA presenting with two coexisting rare symptoms of linear neck pain and prolonged cough.

## Case presentation

A 28-year-old Japanese woman with a history of ulcerative colitis (UC) presented to our hospital with left neck pain, fever, and cough. She was diagnosed with UC when she started having haematochezia six months before her presentation and was initiated on mesalazine. Since then, the frequency of haematochezia has decreased. The symptoms of neck pain and fever started five months earlier than this presentation and improved within one month of nonsteroidal anti-inflammatory drug (NSAID) treatment. At that time, she was diagnosed with viral lymphadenitis. However, her symptoms recurred one month prior to her latest presentation to the hospital, together with prolonged dry cough. The pain was located in her left anterior neck; it occurred as random episodes of constant tingling pain with fluctuations and was alleviated by NSAIDs. The pain was occasionally severe enough to wake her up at night. She denied any aggravating factors or pain in the right side of her neck.

Her vital signs were as follows: blood pressure of 104/66 mmHg and 90/61 mmHg in her right and left arms, respectively; heart rate, 96 beats/min; respiratory rate, 16 breaths/min; and temperature, 36.8℃. On physical examination, she had bilateral carotid bruits and mild linear pulsatile tenderness on her left anterior neck along the carotid artery. Additionally, the left radial artery had a weak pulse with radio-radial delay. No right neck tenderness, thyroid tenderness, or lymphadenopathy was noted. The extremities were warm in all four limbs, and no supraclavicular or infraclavicular bruit was noted. Upon eliciting a further history, she complained of exertional pain on her left arm, especially while she was showering her hair using her left hand or typing on the keyboard at work. She had been on sick leave for a week before her presentation due to her neck pain and exertional pain in her left arm. A complete blood count revealed a low haemoglobin level at 9.4 mg/dL (normal range: 13.7-16.8 g/dL) and a high platelet count at 448 × 10^3^/μL (normal range: 158-348 × 10^3^/μL). There was no leucocytosis. The basic metabolic panel was unremarkable. Additional laboratory tests revealed an elevated erythrocyte sedimentation rate (ESR) at 66 mm/h (normal range: 3-15 mm/h) and C-reactive protein (CRP) level at 13.3 mg/dL (normal range: 0-0.14 mg/dL). Thyroid-stimulating hormone was within normal limits.

**Figure 1:**
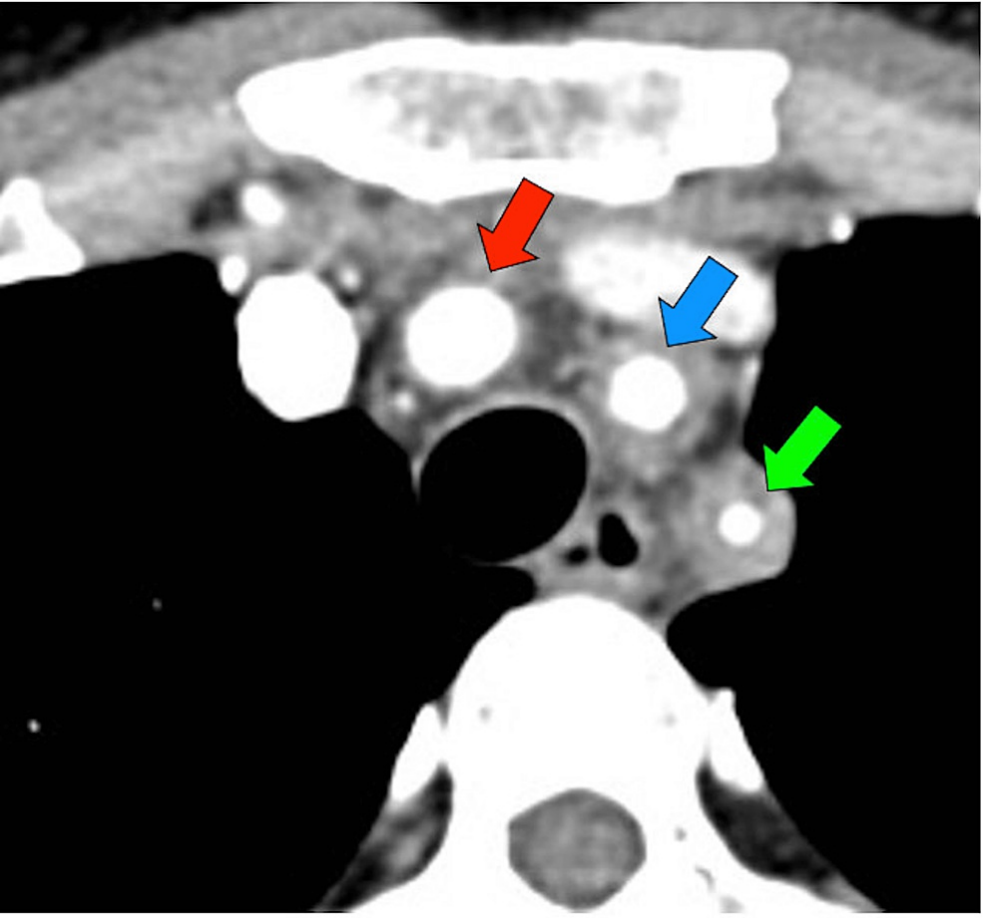
Contrast-enhanced computed tomography scan (axial view) exhibiting wall thickening in the brachial (red arrow), left common carotid (blue arrow), and left subclavian (green arrow) arteries.

**Figure 2:**
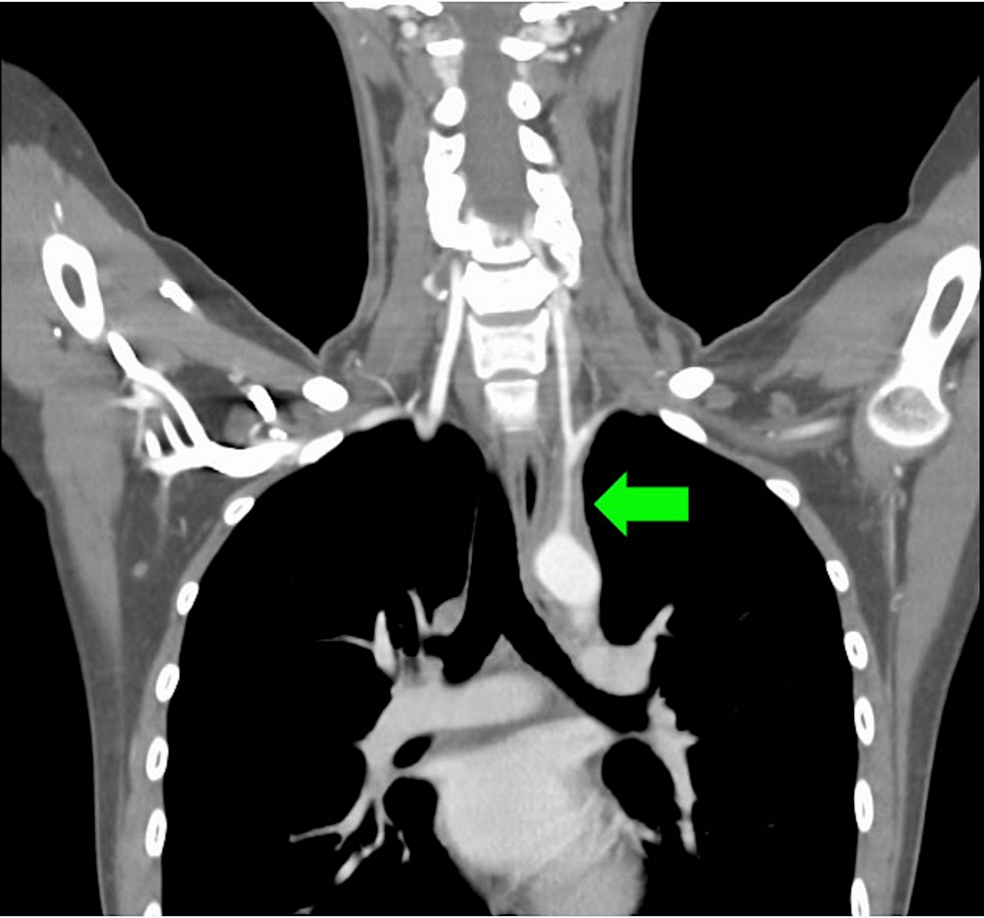
Contrast-enhanced computed tomography scan (sagittal view) exhibiting wall thickening in the left subclavian (green arrow) artery and its branches.

We suspected large vessel vasculitis because she had blood pressure discrepancy between her arms, carotid artery bruit and tenderness, weak unilateral radial pulses, arm claudication, and prolonged recurrent fever with inflammation. Therefore, we conducted a contrast-enhanced computed tomography, which revealed arterial wall thickening involving the aortic arch, both common carotid arteries, and the left subclavian artery (Figures 1, 2). Based on the American College of Rheumatology (ACR) classification criteria, we diagnosed the patient with TA. She was started on 50 mg of prednisone (1 mg/kg) per day for four weeks. All the symptoms improved; thus, the prednisone dose was gradually reduced to 20 mg, and then kept as a maintenance dose. Fifteen months after the initiation of treatment, her neck pain recurred; therefore, methotrexate was prescribed in addition to prednisone. Her symptoms steadily improved; thus, prednisone was gradually reduced to 10 mg per day. Since starting methotrexate, she did not develop any symptom exacerbation; her laboratory examination results, including those of CRP levels and ESR, were stable; hence, both prednisone and methotrexate were completely tapered off 56 weeks after her first presentation. During the follow-up, the patient was determined to be HLA-B52-positive.

## Discussion

We describe a case of a TA who presented with chief complaints of linear neck pain and prolonged cough with a delayed onset of treatment. The diagnosis of TA can be very challenging, especially when rare symptoms coexist. This is because the manifestation of TA greatly varies depending on the vascular involvement and the degree of disease progression. The most common symptoms seen in TA are constitutional symptoms, including fever and weight loss [1]. Arthralgias or myalgias have also been reported as common symptoms, which are present in about 40% of the cases [3]. These symptoms are observed from an early stage, reflecting acute non-specific inflammatory features.

Neck pain and cough usually occur in the late stages of TA in 9.7% [1] and 1.5% [2] of patients, respectively, when arterial insufficiency due to inflammatory vessel wall thickening develops. The mechanism of cough in TA is not exactly understood; one hypothesis is that vessel inflammation stimulates the cough reflex through the affected adjacent vagus nerve [2]. In this case, the co-existence of two rare symptoms which mimicked upper respiratory symptoms delayed the diagnosis. Although her unspecific symptoms can easily be misdiagnosed, the recurring exacerbation of symptoms warranted careful attention to a focused physical examination; this ultimately revealed a blood pressure discrepancy between her arms (> 10 mmHg), and carotid artery bruit, and weak unilateral radial pulses. The patient met four of the six criteria required in the ACR classification criteria and was diagnosed with TA [4].

The link between UC and TA has also been reported in HLA-B52-positive patients like our case [5, 6]. Previous studies have shown that the prevalence of UC among patients with TA was 6.4% and the prevalence of TA in UC patients was 0.21% [5]. A previous review of 32 patients who developed TA and UC revealed that these conditions predominantly developed in females (male to female ratio 1:2) and were reported more in Asian countries (25 cases in Japan, two in India, and one each in Pakistan and Sri Lanka), with a mean age at onset of either condition of 25.6 years (15-56 years) [6]. In this review, UC was antecedent to TA in 21 cases, TA was antecedent to UC in five cases, and the patients in six cases developed both conditions simultaneously [6]. In our case, the patient started complaining of neck pain, which is a late-stage symptom, one month after the diagnosis of UC. The patient may already have developed unspecific early symptoms at the time of UC onset. The high prevalence of these conditions in the Japanese population is considered to be due to the high prevalence of the HLAB52 allele in Japan [7].

Our case underlines the importance of knowing neck pain and cough as uncommon presentations of TA; these symptoms may be overlooked and undervalued by physicians as presentations of TA. This case also highlights that patients with UC are at a higher risk of developing TA compared to the general population.

## Conclusions

The diagnosis of TA can be challenging due to its unspecific symptoms. It is important to recognise neck pain and cough as a rare presentation of TA. This case also highlights that patients with a history of UC are at risk of consequently developing TA, another rare condition if they are positive for HLA-B52.
